# An Ensemble-Based AI Approach for Continuous Blood Pressure Estimation in Health Monitoring Applications

**DOI:** 10.3390/s25154574

**Published:** 2025-07-24

**Authors:** Rafita Haque, Chunlei Wang, Nezih Pala

**Affiliations:** 1 Electrical and Computer Engineering Department, Florida International University, Miami, FL 33174, USA; rhaqu004@fiu.edu; 2 Mechanical and Aerospace Engineering Department, University of Miami, Miami, FL 33136, USA; wangc@miami.edu; 3 Terabrum LLC, Fort Lauderdale, FL 33331, USA

**Keywords:** blood pressure (BP), arterial pulse waveforms (APWs), non-invasive BP estimation, cardiovascular health monitoring, machine learning, AI-augmented diagnostics, wearable medical technologies

## Abstract

Continuous blood pressure (BP) monitoring provides valuable insight into the body’s dynamic cardiovascular regulation across various physiological states such as physical activity, emotional stress, postural changes, and sleep. Continuous BP monitoring captures different variations in systolic and diastolic pressures, reflecting autonomic nervous system activity, vascular compliance, and circadian rhythms. This enables early identification of abnormal BP trends and allows for timely diagnosis and interventions to reduce the risk of cardiovascular diseases (CVDs) such as hypertension, stroke, heart failure, and chronic kidney disease as well as chronic stress or anxiety disorders. To facilitate continuous BP monitoring, we propose an AI-powered estimation framework. The proposed framework first uses an expert-driven feature engineering approach that systematically extracts physiological features from photoplethysmogram (PPG)-based arterial pulse waveforms (APWs). Extracted features include pulse rate, ascending/descending times, pulse width, slopes, intensity variations, and waveform areas. These features are fused with demographic data (age, gender, height, weight, BMI) to enhance model robustness and accuracy across diverse populations. The framework utilizes a Tab-Transformer to learn rich feature embeddings, which are then processed through an ensemble machine learning framework consisting of CatBoost, XGBoost, and LightGBM. Evaluated on a dataset of 1000 subjects, the model achieves Mean Absolute Errors (MAE) of 3.87 mmHg (SBP) and 2.50 mmHg (DBP), meeting British Hypertension Society (BHS) Grade A and Association for the Advancement of Medical Instrumentation (AAMI) standards. The proposed architecture advances non-invasive, AI-driven solutions for dynamic cardiovascular health monitoring.

## 1. Introduction

A comprehensive report by the World Health Organization (WHO) reveals that cardiovascular disease (CVD) continues to dominate as the primary cause of mortality worldwide. In 2016, it was responsible for an estimated 17.9 million deaths, constituting 31% of global fatalities [1]. Timely appraisal and mitigation strategies could appreciably reduce the incidence and fatal outcomes of cardiovascular diseases. As a critical determinant of cardiovascular health, blood pressure is inextricably linked to the progression of CVD. BP monitoring serves as an essential tool in preempting fatal cardiovascular events. Beyond CVD, continuous blood pressure monitoring offers significant clinical value across a range of other health domains. In neurology, it aids in detecting conditions such as stroke, transient ischemic attacks (TIAs), and autonomic dysfunction by revealing abnormal BP fluctuations [2]. In endocrinology and metabolic disorders, it supports early diagnosis of secondary hypertension due to conditions like pheochromocytoma and thyroid dysfunctions [3]. Continuous monitoring is also vital in managing pregnancy-related hypertensive disorders such as preeclampsia [4]. In sleep medicine, nocturnal BP surges have been associated with obstructive sleep apnea, making around-the-clock measurement a useful screening adjunct [5]. Moreover, BP variability is increasingly recognized as a physiological marker of chronic stress and anxiety, offering potential utility in mental health assessment [6]. These applications highlight the broader role of BP as a dynamic biomarker that reflects the state of multiple physiological systems, reinforcing the importance of continuous monitoring in both acute and chronic care settings.

**Figure 1:**
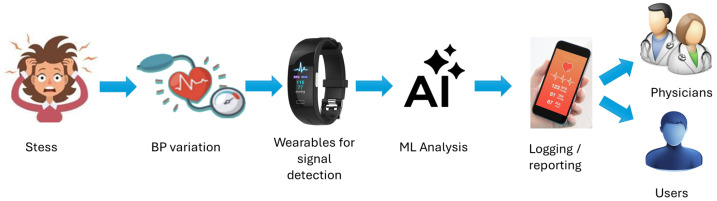
Schematics of AI-enabled system for stress detection via BP monitoring.

Following these clinical insights, Figure 1 illustrates a stress detection system that leverages wearable sensors to continuously monitor variations in blood pressure (BP) and stress-related physiological changes. BP, a dual-value metric typically recorded as 120/80 mmHg, provides critical insights into cardiovascular function—systolic pressure indicating heart contractions and diastolic pressure showing arterial pressure during rest. While heart rate and oxygen saturation are often measured, they alone do not provide a complete picture of cardiovascular status. This system integrates wearable sensors that track BP fluctuations, transmitting data to a smart device where an embedded Stress Detection System (SDS) analyzes these patterns to assess stress levels. Such assessments are essential because stress can significantly influence BP, even when heart rate changes are minimal. By incorporating pulse wave velocity (PWV) and advanced AI-based algorithms like the proposed Fuzzy-Assisted Petri Net (AI-FAS), the system enhances the accuracy of stress evaluation. This technology empowers users with timely insights and promotes proactive stress management, reinforcing the role of continuous BP monitoring in both physical and mental healthcare [7].

**Figure 2:**
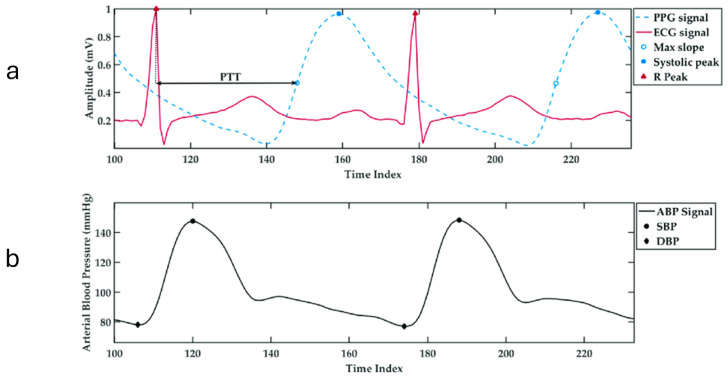
(**a**) The temporal alignment of ECG and PPG signals showing the pulse transit time (PTT), with the R-peak marked on the ECG and the maximum slope and systolic peak marked on the PPG waveform. (**b**) The corresponding arterial blood pressure (ABP) waveform showing systolic blood pressure (SBP) and diastolic blood pressure (DBP) points across time [11].

The commonly used cuff-based devices for automated blood pressure assessment exhibit significant limitations in efficiency. Such devices typically take up to two minutes to obtain systolic and diastolic readings, a time-consuming process reducing their effectiveness in wearable devices for continuous monitoring [8]. To counteract the inefficiencies of conventional methods, neural network-driven regression models have been implemented, effectively shortening BP measurement durations while maintaining strict compliance with the regulatory protocols. These models have achieved commendable success, delivering satisfactory outcomes across diverse clinical settings [9,10]. While the models perform well in operational efficiency and accuracy for continuous BP estimation, their practical use is limited. They rely on extracting physiological data from both electrocardiogram (ECG) and photoplethysmogram (PPG) signals, which can be seen in Figure 2, requiring dual sensors. This increases implementation costs, making them less suitable for widespread adoption.

A significant challenge in deriving physiological parameters from ECG and PPG signals lies in the concept of pulse wave velocity (PWV). PWV refers to the speed at which pressure waves, generated by each heartbeat, travel along the arterial walls. This velocity is known to have a strong correlation with blood pressure, making it a valuable parameter for non-invasive BP estimation. The propagation speed of these waves depends on various arterial properties, such as the elasticity of the arterial wall, its thickness, radius, and the density of blood. One widely used method for estimating PWV involves measuring the time it takes for the pulse wave to travel between two sensors placed at a known distance apart, a measure known as pulse transit time (PTT). However, a major limitation arises when simplifying assumptions are made—such as treating arterial elasticity as a constant. In reality, arterial elasticity varies with pressure, and thus, models that ignore this variation can produce inaccurate results. More advanced approaches attempt to relate PTT to blood pressure using models that account for the pressure-dependent stiffness of arteries. While these models are theoretically sound, their practical use is limited by the difficulty of accurately measuring patient-specific parameters, making them less feasible for widespread clinical application [8].

**Figure 3:**
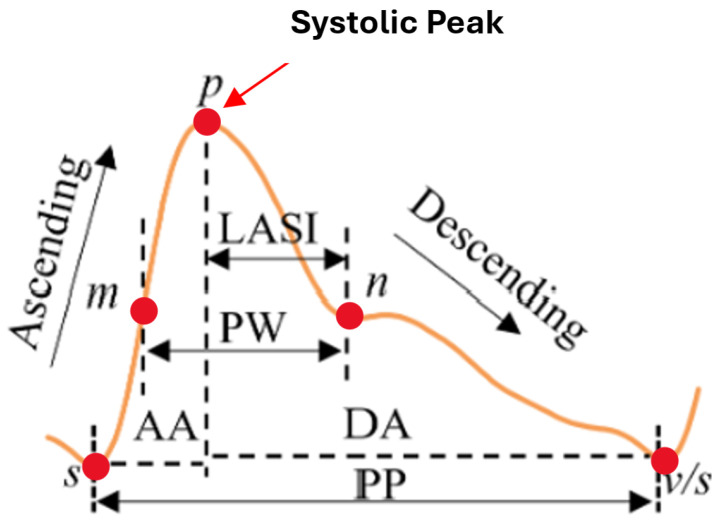
PPG waveform with key feature annotations [11].

Another approach to blood pressure estimation involves constructing a morphological feature model using PPG signals, which requires high-quality data with high sampling rates and precision but is highly susceptible to noise. Integrating ECG and PPG signals, commonly used in clinical settings, enhances estimation accuracy. Regression methods such as SVM, linear regression, [12,13] regression trees, and Random Forests have demonstrated effectiveness, with advanced models like GA-SVR, which combines genetic algorithms and SVM, further improving predictions. Recent studies highlight deep neural networks (DNNs) for capturing complex nonlinear relationships, with frameworks like DBN-DNN showing promising results. The literature reports up to 65 morphological features of pulse waveforms to estimate SBP and DBP [8]. From this pool, 11 key PPG-derived features were selected based on their physiological significance and statistical robustness. Timing-related features such as ascending time, descending time, and pulse width were included to represent different phases of the pulse wave, each influenced by arterial stiffness—a major determinant of blood pressure variability [8,14]. Rate-based features, including pulse rate and pulse intensity rate, reflect heart activity and peripheral perfusion, both of which are closely tied to cardiovascular function [15]. Slope features like ascending slope and descending slope provide insights into the velocity of pressure changes within the arteries, correlating with vascular resistance and elasticity [16]. Area-based features, such as ascending area and descending area, capture volumetric changes in blood flow during a pulse cycle [17]. Finally, intensity variation features—Ascending Intensity Difference and Descending Intensity Difference—were included to quantify amplitude fluctuations in the waveform, often linked to vascular tone and pressure dynamics [18]. As illustrated in Figure 3, critical intervals including the systolic upstroke time, diastolic time, pulse width (PW), pulse period (PP), and late systolic area index (LASI) offer valuable insights into BP variation demonstrating strong potential for accurate non-invasive BP monitoring [8,11].

In this study, we propose an ensemble-based model designed to accurately predict SBP and DBP using features extracted from PPG signals. The model combines the strengths of multiple advanced machine learning techniques to improve prediction accuracy and robustness. Specifically, we utilize Tab-Transformer embeddings to effectively handle tabular data, integrating this with powerful algorithms like CatBoost, XGBoost, and Gradient Boosting. Each of these algorithms brings unique advantages to the ensemble, enhancing the overall performance by leveraging their ability to model complex relationships in the data. By combining these methods, the proposed model ensures reliable and efficient blood pressure estimation, making it suitable applications in health monitoring systems.

## 2. Materials and Methods

**Figure 4:**
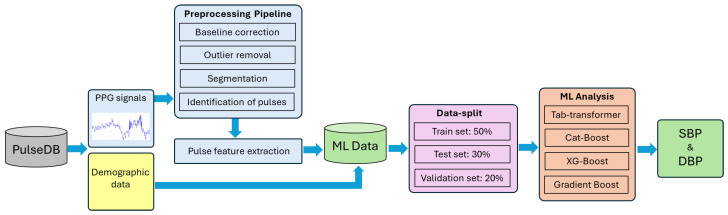
Schematic representation of experimental workflow.

The main flow of the proposed model is schematically shown in Figure 4 and consists of preprocessing, a feature extractor, and an ensemble-based model predictor. Since raw PPG signals may contain noise and baseline drift, preprocessing is essential for effective feature extraction. This section provides a detailed explanation of each component, followed by a summary.

### 2.1. Data Source

**Figure 5:**
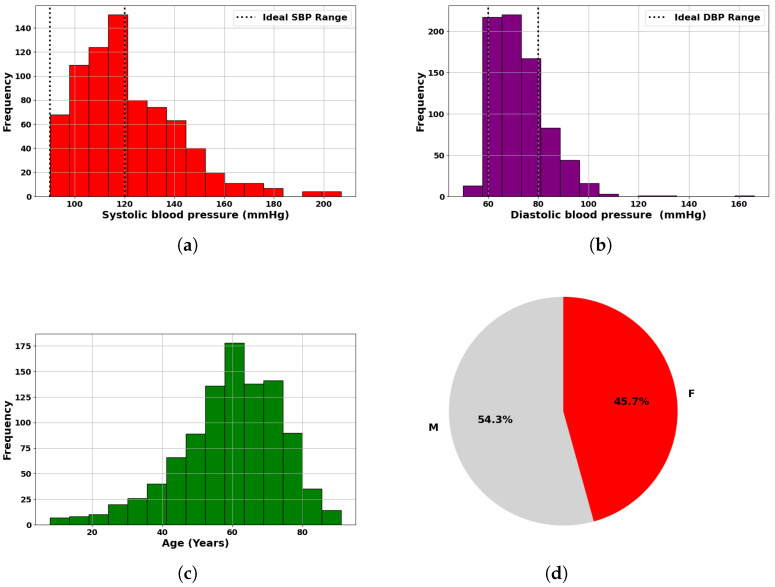
Statistical overview of dataset used in blood pressure analysis: (**a**) Histogram of measured SBP, highlighting ideal systolic range. (**b**) Histogram of measured DBP, indicating ideal diastolic range. (**c**) Age distribution of subjects. (**d**) Gender distribution of subjects.

PulseDB [19] is a dataset designed for benchmarking blood pressure (BP) estimation models while adhering to standardized testing protocols. It comprises 5,245,454 high-fidelity 10 s segments of synchronized ECG, PPG, and arterial blood pressure (ABP) waveforms from 5361 subjects. Curated from the matched subset of the MIMIC-III waveform database and the Vital DB database, it totals 14,570 h of recordings. The data is organized into 5361 MATLAB files, each linked to an individual subject, and includes metadata such as subject details, ECG R-peaks, and PPG systolic peaks, enabling thorough analysis and robust model development [19]. Additionally, the Pulse-DB [19,20] dataset provides demographic features such as age, gender, height, weight, and BMI. These attributes improve model accuracy by enabling personalized BP predictions. The dataset includes segment-averaged systolic and diastolic BP values, essential for training supervised learning models. To ensure diversity and signal quality, ten-second waveform segments were extracted from 1000 subjects, preserving variation in age, gender, and health status. Summary statistics in Table 1 show broad ranges in SBP (50–207 mmHg), DBP (30–172 mmHg), and age (8–91 years), reflecting a mix of normotensive and hypertensive individuals. Figure 4 illustrates dataset variability: Figure 5a,b present SBP and DBP distributions around clinical norms with clear outliers; Figure 5c shows a wide age distribution centered near 60 years; and Figure 5d indicates a nearly balanced gender ratio (54.3% male, 45.7% female). Together, these features were used to develop robust, generalizable models for non-invasive BP monitoring.

### 2.2. Preprocessing

**Figure 6:**
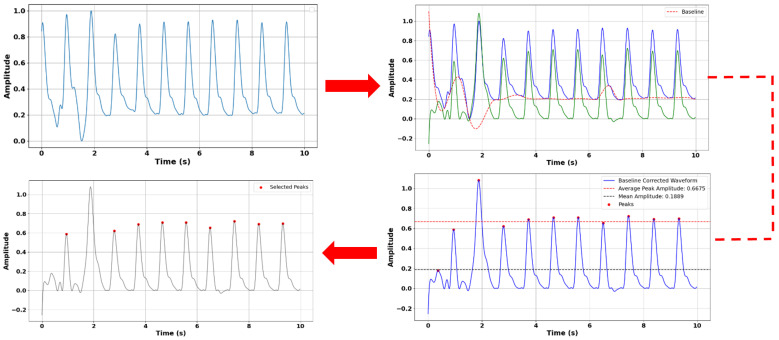
Preprocessing of PPG signal for reliable feature extraction. Top left: Original PPG signal. Top right: Baseline correction with the original PPG signal (blue), baseline (red) and baseline corrected signal (green). Bottom right: Maxima detection. Bottom left: Final pulses used in feature extraction.

Before estimating blood pressure using PPG signals, we applied a comprehensive preprocessing pipeline to enhance signal quality and ensure accurate feature extraction (Figure 6). Initially, although Pulse-DB (Vital) provides pre-filtered PPG signals using a Chebyshev-II band-pass filter (0.5–8 Hz), we further improved data reliability by applying the Interquartile Range (IQR) method to detect and remove outliers. Pulses with peak amplitudes and pulse periods beyond 1.5 times the IQR from the first or third quartile were excluded to maintain signal consistency. We then performed baseline correction to eliminate low-frequency drift by identifying local minima as baseline points and modeling the drift using a cubic spline function, which was subtracted from the original waveform to produce a clean signal. Although PulseDB provides pre-filtered signals using a 0.5–8 Hz Chebyshev-II band-pass filter, we observed residual baseline wander and local low-frequency trends in certain pulse segments. Therefore, we implemented baseline correction using cubic spline interpolation to further enhance signal clarity. This step addressed subtle nonlinear drifts not fully removed by the standard band-pass filter, particularly around local minima and during pulse transitions. IQR-based outlier removal for amplitude and pulse width also helped filter out non-physiological or deformed pulse shapes (e.g., absent or distorted dicrotic notches) during feature extraction to ensure waveform quality and consistency in training. Feature point detection was carried out on the corrected signal, where systolic peaks and diastolic troughs were identified using a minimum distance threshold to ensure physiological accuracy and reduce false detections. Subsequently, amplitude statistics were analyzed by calculating average peak and mean values, selecting only pulses with amplitudes between the mean and 20% of the average peak for feature extraction. From these selected pulses, we extracted 11 morphological features: ascending time, descending time, pulse width, pulse rate (BPM), pulse intensity rate, ascending and descending slopes, ascending and descending areas, and intensity differences—at a sampling rate of 125 Hz. To enhance model accuracy and generalizability, we also integrated demographic information age, gender, height, weight, and BMI, enabling personalized insights into pulse waveform characteristics [8,21,22,23,24,25].

### 2.3. Overview of Proposed Network Architecture

The proposed model architecture integrates both deep learning and ensemble learning approaches to accurately estimate SBP and DBP blood pressure. At its core, a Tab-Transformer is employed, featuring an embedding layer (Dim = 64, Depth = 6, Heads = 8, Dropout = 0.2) followed by multiple transformer blocks with ReLU activation and MLP hidden layers (4, 2). Alongside this, three ensemble regression models—CatBoost, XGBoost, and LightGBM—are individually optimized for SBP and DBP prediction. These models are fine-tuned with hyperparameters such as 300–1000 estimators or iterations, learning rates between 0.01 and 0.1, and maximum depths ranging from 3 to 10. For XGBoost, additional tuning is performed for subsample and column sample rates. The outputs from all three models are then fused using a concatenation-based fusion block and passed to a gradient boosting meta-model configured with 200 estimators, a 0.05 learning rate, and a maximum depth of 4. To ensure robustness, the complete architecture is validated through 5-fold cross-validation with a fixed random state of 42 for consistent performance measurement.

### 2.4. Experimental Details

We used a unique estimation framework that integrates deep contextual embeddings from the Tab-Transformer model [26,27,28,29,30,31] with ensemble learning algorithms—CatBoost [32], XGBoost [33], and LightGBM [34]—to model the nonlinear relationships in pulse waveform-derived features. CatBoost was selected for its ability to handle categorical data efficiently using ordered boosting and symmetric trees, minimizing overfitting and prediction shift. XGBoost uses regularized gradient boosting with second-order optimization, while LightGBM adopts a histogram-based, leaf-wise growth strategy for faster training and improved accuracy on large datasets. Hyperparameters such as tree depth, learning rate, and the number of estimators were fine-tuned using Optuna to maximize model performance. To enhance feature representation, we employed Tab-Transformer, a transformer-based architecture specifically designed for tabular data, which differs from traditional models by applying multi-head self-attention to contextualized embeddings of categorical variables. These embeddings were combined with normalized continuous features and passed through a multilayer perception, producing high-level representations that were stacked alongside base model predictions. The stacked ensemble, using a Gradient Boosting Regressor [35] as the meta-learner, was trained on both raw and engineered features, including area ratios, polynomial terms, and intensity differences. We applied RobustScaler for normalization and used mutual information-based feature selection to improve generalization.

To ensure model robustness, 5-fold cross-validation (KFold, n_splits = 5, shuffle = True, random_state = 42) is implemented, where the dataset is split into five subsets, iteratively using four folds for training and one for validation. This approach ensures better generalization, prevents overfitting, and allows each sample to contribute to both training and validation. During each fold, the Tab-Transformer processes categorical and continuous features, followed by ensemble models (CatBoost, XGBoost, and LightGBM) trained separately for SBP and DBP. Predictions from these models are stacked and passed through a Gradient Boosting meta-regressor, further refining final outputs by learning residual errors. The combination of transformer-based feature encoding and tree-based boosting methods enhances the model’s ability to capture intricate nonlinear dependencies.

The proposed hybrid regression model was implemented in Python (Python Software Foundation, v3.10.14) using libraries like PyTorch (PyTorch/Linux Foundation, v2.3.0), Scikit-learn, SciPy, and Tab-Transformer. Training and testing were conducted on Google Colab with a Python 3 runtime and T4 GPU for efficient computation. For local development, an ASUS laptop with an Intel Core i9 processor, NVIDIA RTX 3050 GPU, 16 GB RAM, and 1TB SSD was used. MATLAB (Mathworks, Inc. version R2023b) supported data analysis and validation, ensuring seamless integration between local and cloud environments. This setup provided robust and efficient execution of the ensemble learning methodology [8].

## 3. Results

Performance evaluation demonstrates high predictive accuracy, achieving R^2^ scores of 0.9393 (SBP) and 0.9181 (DBP), with RMSE values of 5.56 mmHg (SBP) and 3.90 mmHg (DBP) and MAE values of 3.87 mmHg (SBP) and 2.50 mmHg (DBP). These results affirm the robustness of our stacking-based learning approach, effectively capturing intricate temporal and nonlinear relationships while mitigating overfitting.

Based on the learning curves, we can see how the proposed model—using XGBoost—performs for both SBP and DBP blood pressure predictions. On the left, the SBP curve shows a sharp drop in RMSE within the first 200 iterations, indicating that the model learns quickly. After about 300 iterations, the curve flattens, suggesting convergence. On the right, the DBP curve follows a similar trend but reaches a lower RMSE, showing that the model is slightly more effective at learning DBP patterns. Overall, the proposed model demonstrates efficient training and reaches optimal performance within 500 iterations, with no sign of overfitting.

**Figure 7:**
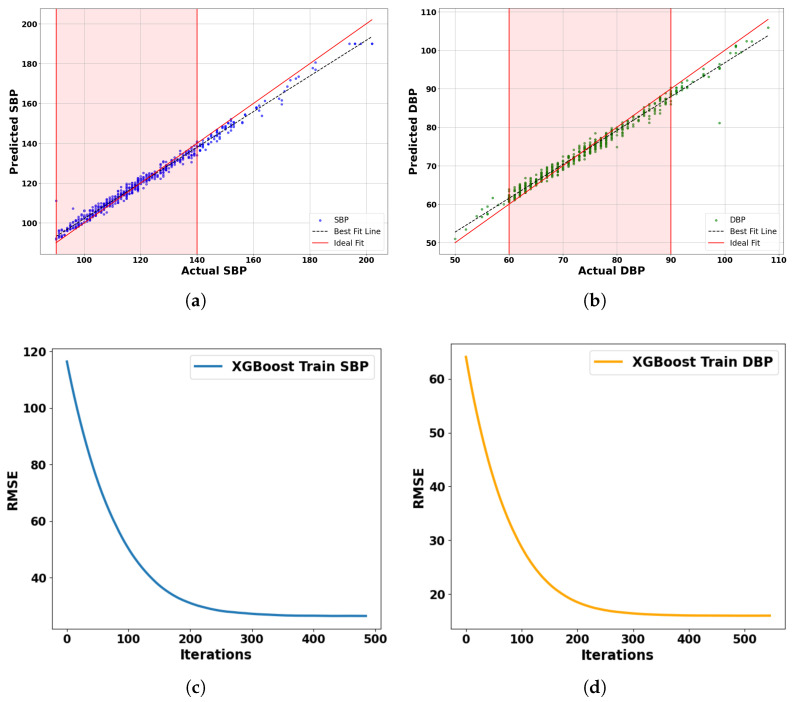
Comparative analysis of blood pressure prediction models using test set comprising 150 subjects: (**a**) Predicted vs. actual SBP. (**b**) Predicted vs. actual DBP. (**c**) Learning curve for SBP. (**d**) Learning curve for DBP.

To comprehensively evaluate the model’s predictive performance, we generated scatter plots comparing actual versus predicted values for both SBP and DBP, as shown in Figure 7a,b. Each plot includes an ideal fit line (red) and a best fit regression line (black dashed). The regression lines closely follow the ideal fit line (y = x), with average minimum deviations of just 1.45 mmHg for SBP and 0.85 mmHg for DBP. The shaded regions indicate clinically significant blood pressure ranges (SBP: 90–140 mmHg, DBP: 60–90 mmHg), where the model performs exceptionally well. These outcomes confirm that the model meets and exceeds clinical standards such as AAMI and BHS for clinical applications.

**Figure 8:**
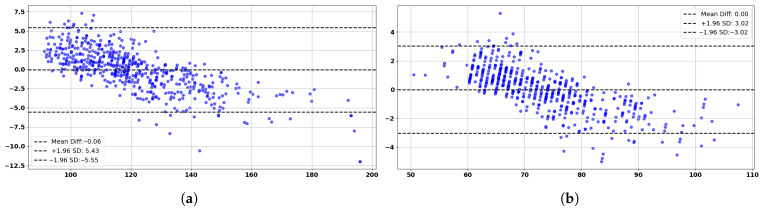
Bland–Altman analysis based on the test set of 150 subjects: (**a**) Predicted vs. actual SBP. (**b**) Predicted vs. actual DBP.

These promising results are further corroborated by the Bland–Altman analysis, as shown in Figure 8a,b, which provide additional insights. The analysis shows strong agreement between predicted and actual blood pressure values, as demonstrated by the Bland–Altman analysis for SBP and DBP measurements and predictions. For SBP, the mean difference is −0.03 mmHg with 95% limits of agreement (±1.96 standard deviations) ranging from −5.45 mmHg to +5.40 mmHg, indicating negligible bias and clinical compliance with AAMI standards. Similarly, the DBP Bland–Altman plot reveals a mean difference of −0.03 mmHg, with limits of agreement between −3.19 mmHg and +3.13 mmHg. Both plots confirm that the majority of data points fall within the clinically acceptable range, with no significant systematic over- or underestimation. The results validate the reliability and consistency of the proposed model across a wide range of blood pressure values, supporting its suitability for continuous, non-invasive cardiovascular monitoring applications.

### 3.1. Clinical-Grade Accuracy: Compliance with AAMI and BHS Standards

Our model achieves clinical-grade accuracy in blood pressure estimation, meeting both AAMI and BHS standards. It results in low MAE (3.87 mmHg for SBP, 2.50 mmHg for DBP) and SD (5.61 mmHg for SBP, 3.88 mmHg for DBP), surpassing AAMI benchmarks. Additionally, it attains BHS Grade A classification, with over 85% of SBP and 89% of DBP predictions falling within ±5 mmHg. These results confirm the model’s robustness, reliability, and suitability for noninvasive, real-time BP monitoring in clinical settings [8,11].

### 3.2. Consistent Performance Across Diverse Subject Groups

To test the robustness of our model across diverse subject groups, we selected sub-groups from the dataset as shown in Table 2 and preformed prediction analysis. Table 3 evaluates its predictive accuracy across dataset partitions. High R^2^ scores (e.g., 0.9393 for SBP, 0.9181 for DBP in Group 1) confirm the model’s ability to explain BP variance, while low MAE values (e.g., 1.55 mmHg SBP, 1.43 mmHg DBP in Group 4) highlight its precision in specific cohorts. Across gender-based subsets (Group 2: Female, Group 3: Male), the model maintains stable RMSE values (5.73 mmHg SBP, 3.65 mmHg DBP), showcasing strong generalization. The model complies with AAMI standards, with SBP SD of 5.61 mmHg and DBP SD of 3.88 mmHg, ensuring clinically acceptable error margins. Transformer-driven feature embeddings further enhance adaptive learning across physiological variations. These results validate the partitioning strategy, confirming high accuracy and clinical reliability across population subsets [23,25].

### 3.3. Comparative Analysis: Individual Models vs. the Integrated Model

To systematically evaluate the effectiveness of our proposed approach, we conducted a comprehensive comparison of each individual model utilized in the study, as outlined in Table 4. This includes CatBoost, XGBoost, LightGBM, and Tab-Transformer models, each trained independently using the same set of selected features. Their performance in predicting systolic (SBP) and diastolic (DBP) blood pressure was assessed using Mean Absolute Error (MAE) and R^2^ score. Notably, the Tab-Transformer demonstrated the highest R^2^ for DBP (0.9312), while CatBoost achieved the lowest MAE for SBP (3.88 mmHg), reflecting superior accuracy in systolic pressure prediction. LightGBM also stood out with the highest R^2^ score for DBP (0.9251), highlighting its capability in diastolic pressure estimation. Building upon the strengths of these individual models, we developed an ensemble-based hybrid model that strategically combines their outputs through a meta-learning framework. This integration resulted in the highest R^2^ for SBP (0.9393) and the lowest MAE for DBP (2.50 mmHg), indicating a well-balanced and superior overall performance. While individual models showed specific strengths, our proposed ensemble model stands out by demonstrating unmatched robustness and consistency across both systolic and diastolic blood pressure parameters. These findings underscore the power of model fusion, confirming that the integration of multiple learning algorithms can significantly enhance predictive reliability and generalizability in non-invasive blood pressure estimation.

### 3.4. Comparative Analysis: Our Model vs. Other ML Approaches

We tested the blood pressure prediction performance of three powerful models and compared them in Table 5. Random Forest, a trusted traditional model, delivers decent performance with R^2^ scores of 0.8804 (SBP) and 0.8946 (DBP), though it struggles with higher MAE values (5.8936 and 3.4093 mmHg). Then comes XGBoost, the heavyweight of precision, presenting better R^2^ scores of 0.8976 (SBP) and 0.8980 (DBP) and MAEs of 5.6991 and 5.3725 mmHg. Yet, it is our Hierarchical Transformer-Boosted Model (HTBM) that strikes the perfect balance. With R^2^ scores of 0.9393 (SBP) and 0.9181 (DBP) and impressively low MAEs of 3.87 and 2.50 mmHg, HTBM does not just predict—it explains. While LS Boosting edges ahead in raw accuracy, HTBM triumphs with its unmatched interpretability, making it a powerful and practical solution for non-invasive, real-world blood pressure monitoring [36,37,38,39,40].

To evaluate the performance of our proposed model against existing methods, we compared it with several state-of-the-art PPG-based blood pressure estimation models, as shown in Table 6. Among the models evaluated using the MIMIC II dataset, the BiLSTM + LSTM + attention architecture demonstrated strong performance, achieving Mean Absolute Errors (MAEs) of 4.51 mmHg for SBP and 2.6 mmHg for DBP. U-Net and spectro-temporal ResNet showed moderate accuracy, while traditional approaches like SVM exhibited the highest error rates, with MAEs of 12.38 mmHg (SBP) and 6.34 mmHg (DBP). Our proposed model—trained on the Pulse-DB dataset with 1000 subjects—outperformed all other methods by achieving the lowest MAEs: 3.87 mmHg for SBP and 2.50 mmHg for DBP. These results confirm the enhanced accuracy and robustness of our integrated approach for non-invasive blood pressure monitoring.

### 3.5. Constraints and Proposed Solutions

The developed model underwent validation exclusively on the Pulse-DB dataset, thereby restricting its evaluation to data derived from a single institutional source. This limitation inherently constrains the model’s generalizability and diminishes its potential applicability across heterogeneous clinical environments. Furthermore, the model’s operational robustness under dynamic, real-world conditions—particularly those involving user mobility—remains insufficiently investigated. Given that motion artifacts introduced during physical activity may distort sensor signal fidelity, the absence of a comprehensive motion-resilience assessment represents a critical gap.

To enhance the translational potential of this approach, future research should emphasize rigorous external validation leveraging multicenter datasets, systematically evaluate artifact resilience under ambulatory conditions, and substantiate the model’s clinical utility through prospective real-world trials [45].

## 4. Conclusions

We presented a Hierarchical Transformer-Boosted Model (HTBM) for accurate SBP and DBP estimation using PPG signals. By incorporating both physiological and demographic features from 1000 subjects, the model achieves a balanced performance, with $R^{2}$ scores of 0.9393 (SBP) and 0.9181 (DBP) and MAE values of 3.87 mmHg (SBP) and 2.50 mmHg (DBP).

While XGBoost outperforms HTBM in terms of accuracy, the HTBM model offers greater interpretability, making it a practical option for blood pressure estimation. It reduces prediction errors compared to traditional models like Random Forest, while maintaining competitive accuracy with XGBoost. This model shows promise for non-invasive blood pressure monitoring, meeting high standards of predictive accuracy with minimal error variation across different subject groups, including female and overweight subgroups.

The proposed model can be seamlessly integrated into a smartwatch-like wearable device, in a compact, user-friendly form factor. This integration would involve optimizing the model for low-power edge computing, enhancing computational efficiency, and ensuring seamless data acquisition and transmission. Additionally, expanding the dataset to encompass non-clinical and ambulatory environments would enhance model generalizability, improving robustness against motion artifacts and physiological variability. These advancements will contribute to the development of a scalable, real-world deployable solution for non-invasive BP tracking, making personalized cardiovascular health monitoring more accessible, reliable, and clinically viable in everyday settings.

## Figures and Tables

**Table 1:** Statistical characteristics of blood pressure measurements in experimental database.

Feature	Std Dev	Mean	Median	Min.	Max.
SBP	114.61	23.07	113.0	50	207
DBP	62.73	14.66	62.0	30	172
Age	59.33	14.52	61.0	8	91

**Table 2:** Summary of dataset partitioning.

Group	Number of Subj.	Training Set	Test Set	Validation Set
1	1000	700	150	150
2	900	700	100 (Female)	100
3	900	700	100 (Male)	100
4	270	150	20 (BMI ≥ 25)	100

**Table 3:** Subject-wise evaluation of BP estimation accuracy (proposed model).

Group	$R^{2}$ Score SBP	$R^{2}$ Score DBP	Avg. RMSE SBP	Avg. RMSE DBP	MAE (SBP) (mmHg)	MAE (DBP) (mmHg)
1	0.9393	0.9181	5.56	3.90	3.87	2.50
2	0.9347	0.9276	5.73	3.65	4.07	2.39
3	0.9379	0.9190	5.61	3.86	3.87	2.66
4	0.9411	0.9126	4.52	3.65	1.55	1.43

**Table 4:** A comparison of the performance of the individual models and the integrated model used in this study.

Methods	R^2^ SBP	R^2^ DBP	MAE SBP (mmHg)	MAE DBP (mmHg)
CatBoost	0.9171	0.9192	3.88	2.69
XGBoost	0.9052	0.9128	3.59	3.56
LightGBM	0.9073	0.9251	3.89	2.81
Tab-Transformer	0.9116	0.9120	3.91	3.34
Our proposed ensemble model	0.9393	0.9181	3.87	2.50

**Table 5:** Comparison of our proposed model with other methods on 1000 records.

Methods	$R^{2}$ Score		MAE (mmHg)	
	SBP	DBP	SBP	DBP
Random Forest Regression	0.8804	0.8946	5.8936	3.4093
Extreme Gradient Boosting	0.8976	0.8980	5.6991	5.3725
LS Boosting	0.9502	0.9622	3.93	3.03
Our Hierarchical Transformer-Boosted Model (HTBM)	0.9393	0.9181	3.87	2.50

**Table 6:** A comparison of the performance of the PPG-based BP prediction models reported in the literature with our integrated model used in this study.

Dataset	Methods	Number of Subjects	MAE (mmHg)		BHS Grade	AAMI Status	Ref.
			SBP	DBP			
MIMIC II	Random Forest Regression	441	12.75	6.04	A (DBP), D (SBP)	DBP Pass	[36]
MIMIC II	Long-term Recurrent Convolutional Network	510	9.43	6.88	Grade C	Fail	[41]
MIMIC II	Residual Network with 152 layers	942	12.98	8.78	Fail	Fail	[42]
Queensland and MIMIC II	KNN + Category-Wise Regression Tree	282	7.1	6.0	B (SBP), A (DBP)	Pass	[13]
MIMIC II	U-shaped Convolutional Neural Network	942	5.73	3.45	A(DBP)	DBP Pass	[43]
MIMIC II	BiLSTM + LSTM + Attention	942	4.51	2.60	B (SBP), A (DBP)	Pass	[44]
MIMIC II	Support Vector Machine	1000	12.38	6.34	B (DBP)	Fail	[12]
Pulse-DB-Vital	Our Proposed Model	1000	3.87	2.50	A (SBP, DBP)	Pass	This work

## Data Availability

The dataset used in our article is publicly available at https://github.com/pulselabteam/PulseDB (accessed on 17 July 2025) and is licensed under Creative Commons Public Licenses as indicated in the repository. The processed data used for model training and evaluation may be made available by the corresponding author upon reasonable request and with appropriate institutional approval.
